# Controlled-release potassium sulfate combined with the deep banding method on soybean productivity and soil microbial characteristics

**DOI:** 10.3389/fpls.2026.1904680

**Published:** 2026-07-29

**Authors:** Qianhui Chen, Shuhan Yin, Changzhi Li, Jie Qu, Yongxiang Gao, Qi Chen, Yanli Liu, Chengliang Li, Zhaoming Qu

**Affiliations:** National Engineering Research Center for Efficient Utilization of Soil and Fertilizer Resources, College of Resources and Environment, Shandong Agricultural University, Tai’an, China

**Keywords:** endogenous hormone, fertilization method, potassium fertilizer, potassium use efficiency, soybean yield

## Abstract

Controlled-release potassium sulfate (CPS) and deep banding fertilization are promising strategies for improving crop productivity, but their combined effects on soybean growth remain unclear. In this study, field experiments were performed over two growing seasons (2023–2024) to evaluate how potassium (K) fertilizer types (potassium sulfate and CPS) and fertilization methods (broadcast incorporation and deep banding) influence soil microbial composition, soil enzyme activity, and soybean growth characteristics. The results indicate that deep banding and CPS application consistently increased soybean yield, K use efficiency, and nitrogen use efficiency. Relative to broadcast incorporation, the application methods of deep banding increased soybean yield by 1.1%–2.7% in 2023 and 3.7%–3.8% in 2024, while the application of CPS increased soybean yield by 6.0%–7.7% and 9.6%–9.7% compared with potassium sulfate (PS) application. Additionally, deep banding of CPS increased soil protease, urease, acid phosphatase, and phosphodiesterase activities and improved plant enzyme activities and hormone contents. Microbial analyses showed that CPS combined with the deep banding method (DCPS) enriched several microbial taxa (*Bradyrhizobium*, *Lysobacter*, *Rhizophagus*, and *Funneliformis*) potentially associated with nutrient cycling and was associated with more connected genus-level microbial co-occurrence networks. Structural equation modeling further indicated that microbial diversity, soil fertility, enzyme activity, and plant growth characteristics were jointly linked to soybean productivity. These results suggest that DCPS is a promising K management strategy for improving soybean productivity and nutrient use efficiency in the North China Plain.

## 1 Introduction

Soybean (*Glycine max* (L.) Merr.) serves as a primary leguminous crop across China and plays a foundational role in safeguarding the country’s food security (Yang et al., 2024). As the world’s largest soybean consumer, China reached a national soybean consumption figure of 117 million tons in 2023, as documented in the FAO Corporate Statistical Database (FAO, 2024). Despite its massive domestic consumption demand, China suffers from a stark mismatch between indigenous soybean production capacity and market requirements. In 2023, the nation imported 99.4 million tons of soybeans, translating to a domestic self-sufficiency rate of merely 15% and an external dependence rate of over 80% (Wang et al., 2023). Under this supply-demand constraint, improving soybean production capacity is vital to revitalize China’s soybean industrial chain and further strengthen the resilience of the country’s food security (Wu et al., 2024). Potassium (K) is an essential macronutrient that governs vegetative development and reproductive yield formation in soybean. As a universal activator for a wide array of soil and plant enzymes, K also modulates key nitrogen (N) metabolic processes. It optimizes the activity of critical metabolic enzymes, including nitrate reductase and glutamine synthetase, regulates the N cycling pathway, and facilitates the translocation of photosynthates, thereby indirectly improving N uptake and utilization efficiency in soybeans (Singh and Kataria, 2012). Notably, potassium sulfate (PS) exhibits superior performance over potassium chloride (PC) in enhancing soybean protein synthesis and N utilization efficiency. The superiority stems from sulfur (S) supplementation, a secondary nutrient absent in PC fertilizers. The supplied S further supports nodule-mediated biological N fixation in soybean plants (Rushovich and Weil, 2021). Despite the well-documented agronomic advantages of K fertilization, inappropriate and excessive K input in soybean production commonly triggers soil K fixation. This process drastically diminishes nutrient use efficiency, increases farming input costs, and induces long-term agroecological problems, such as secondary soil salinization and disrupted soil nutrient stoichiometry. Accordingly, optimized K fertilization scheduling is urgently required to reconcile high-yield soybean production, efficient nutrient utilization, sustained soil fertility, and overall agricultural sustainability.

Controlled-release potassium sulfate (CPS) is an eco-friendly K fertilizer product characterized by a potassium release characteristic that matches the potassium requirements of crops. This synchronized nutrient supply mechanism effectively enhances crop K uptake efficiency and minimizes soil K fixation losses (Qu et al., 2024). Controlled-release K fertilizers are widely acknowledged as a sustainable agronomic practice in modern farming systems, capable of simultaneously improving crop productivity and nutrient utilization proficiency. A large number of field trials on major staple crops have consistently demonstrated that controlled-release K fertilizers outperform conventional K sources in yield promotion and nutrient retention efficiency. For example, Yang et al. (2016) observed that compared with PC application, the application of controlled-release potassium chloride (CPC) improved K recovery efficiency by 24.3%–33.8% and increased cotton lint yield by 11.2%–32.1%. In a wheat-based study, Li et al. (2021b) reported that CPC application elevated grain yield by 5.0%–19.7%, enhanced K use efficiency (KUE) by 19.8%–42.7%, and increased net economic returns by 7.2%–28.7%. Additionally, CPC fertilization substantially improved wheat root morphological development, with root volume and surface area increased by 80.2% and 38.8%, respectively, alongside a 19.5% rise in root auxin concentration. Qu et al. (2024) also documented 4.5%–28.7% higher maize yield and 5.9–28.7 percentage points higher KUE under CPC fertilization. Collectively, these field evidence validates the great potential of controlled-release K fertilizers in improving crop yield and nutrient use efficiency while preventing excessive K accumulation in cultivated soils. Nevertheless, existing studies predominantly focus on the nutrient release synchronization of CPC with crop K demand and its yield-enhancing effects (Li et al., 2020; Qu et al., 2020). There remains a distinct research gap regarding how the application of CPS regulates soybean productivity by modulating soil enzyme activity and soil microbial characteristics, which limits our understanding of the mechanisms underlying the use of CPS in soybean production systems.

Beyond fertilizer type, fertilization methods serve as another key determinant of crop growth, nutrient absorption, and final yield formation (Delbaz et al., 2023; Andrade et al., 2023). The agronomic performance of varied fertilization methods on crop productivity and nutrient use efficiency has been extensively validated across diverse cropping systems (Nkebiwe et al., 2016; Paut et al., 2024). Deep banding fertilization, which places concentrated fertilizer strips in subsurface soil layers, has emerged as an optimized and efficient fertilization technique. Cumulative field findings confirm that deep banding reshapes the vertical spatial distribution of soil nutrients and elevates nutrient bioavailability in subsoil horizons (Sharifi et al., 2024; Jiang et al., 2018). Meanwhile, this localized fertilization approach concentrates nutrients within a targeted soil volume, forming a high-concentration nutrient microenvironment in the rhizosphere. It reduces the contact interface between fertilizer particles and bulk soil, thus mitigating nutrient fixation, maintaining a stable and adequate supply of nutrients throughout the growing season, and ultimately promoting nutrient uptake and yield formation in crops (Zhou et al., 2025b). Compared with traditional surface broadcasting, deep banding has been proven to improve yield performance and fertilizer use efficiency in multiple crops, including rice (Li et al., 2021a), oilseed rape (Tenuta et al., 2023), cotton (Bai et al., 2025), wheat (Liu et al., 2022), and maize (Wang et al., 2024). However, current research on deep banding practices mainly concentrates on N and phosphorus (P) fertilizer management. Relevant studies focusing on K fertilizer combined with the deep banding method, particularly in soybean field production, remain limited. It is therefore critical to explore the effects of different K fertilizer types combined with fertilization methods on soybean growth and clarify the underlying mechanisms driving crop nutrient absorption and yield formation.

Optimal selection of K fertilizer types and fertilization methods is an essential prerequisite for stabilizing soil fertility, optimizing nutrient utilization, and maximizing crop yield potential (Di Mauro et al., 2023). While prior studies have confirmed the positive effects of CPC and the benefits of deep banding of N and P fertilizers in agricultural production, the synergistic effects of combined CPS and the deep banding methods have not been investigated in soybean cropping systems. The present study aimed to systematically evaluate the interactive effects of CPS application and deep banding method on soybean growth and productivity. We further elucidated the independent and combined contributions of plant physiological characteristics (antioxidant enzyme activities and endogenous hormone contents) and soil ecological properties (microbial characteristics and enzyme activities) to soybean yield formation. The specific research objectives were: (1) to clarify the responses of soybean yield and growth traits to different K fertilizer types and fertilization methods; (2) to quantify the relative contributions of plant physiological indicators, soil enzyme activities, and soil characteristics to soybean productivity, and (3) to identify the optimal combination of fertilization method and K fertilizer type for balancing high soybean yield and efficient nutrient utilization.

## 2 Materials and methods

### 2.1 Experimental site description

A two-year field experiment was conducted during two soybean growing seasons (2023 and 2024) at the “Cidian Farm”, Jining City, Shandong Province, China (35°41′ N, 116°39′ E). This study area is characterized by a temperate continental semi-humid monsoon climate. During the soybean growing season, the mean air temperature was 26.9°C in 2023 and 27.3°C in 2024, while cumulative precipitation was 405.0 and 726.5 mm, respectively (**Supplementary Figure 1**). The soil texture of the 0–20 cm soil layer is Typic Hapli-Udic Argosol according to the Chinese Soil Taxonomy system. The soil pH, soil organic matter, soil inorganic N (IN), available P, and available K (AK) of the cultivated soil layer (0–20 cm) were 8.1 (1:2.5 soil–water suspension), 16.4 g kg^-1^, 40.9 mg kg^-1^, 12.3 mg kg^-1^, and 140.3 mg kg^-1^.

### 2.2 Experimental design

The field experiment was arranged in a randomized complete block design with three biological replicates, consisting of six distinct fertilization treatments: (1) nitrogen-free fertilization with broadcast incorporation of PS at 5 cm soil depth (NNF); (2) potassium-free fertilization (NKF); (3) PS combined with broadcast incorporation method at 5 cm soil depth (BPS); (4) PS combined with deep banding method at 15 cm soil depth (DPS); (5) CPS combined with broadcast incorporation method at 5 cm soil depth (BCPS); and (6) CPS combined with deep banding method at 15 cm soil depth (DCPS). Each plot measured 5 m × 4 m (20 m²), with a 0.5 m buffer zone between adjacent plots.

Soybean cultivar ‘Shanning 16’ was sown at a density of 192,500 plants ha^-1^ in all experimental plots. Each plot contained 11 rows with 35 plants per row, maintaining 45-cm row spacing and 11-cm intra-row spacing, for a total of 385 plants per plot. Sowing was performed on June 15, 2023, and June 16, 2024, with corresponding harvests on October 8, 2023 (116 days after sowing, DAS) and October 5, 2024 (112 DAS). Pre-planting K fertilizer bands (15-cm depth) were manually established between every two soybean rows for deep placement treatments. For the broadcast incorporation treatment, fertilizers were evenly spread on the soil surface and subsequently mixed into the top 5 cm of the soil layer using a rotary tiller.

Three conventional commercial fertilizers were adopted in this study, including urea (46% N), triple superphosphate (46% P_2_O_5_), and conventional PS (54% K_2_O). The CPS (50% K_2_O) was self-prepared by coating conventional PS granules in a rotary drum at the National Engineering Laboratory for Efficient Utilization of Soil Fertility Resources of China. The coating formulation consisted of 0.5% paraffin and 3.5% polyurethane, following the preparation protocol established by Chen et al. (2020). The K release characteristics of the CPS shown in **Supplementary Figure 2** were determined before the experiment using the method of the People’s Republic of China (HG/T 4216-2011). All fertilization practices were implemented one day before sowing in accordance with local standard soybean cultivation management. Urea and triple superphosphate were uniformly broadcast and incorporated into the 0–5 cm soil layer at application rates of 75 kg P_2_O_5_ ha^−1^ and 45 kg N ha^−1^, respectively, with the NNF treatment excluded from nitrogen application. All K-fertilizer treatments received an identical K input of 70 kg K_2_O ha^−1^, except for the NKF control treatment with no potassium input.

### 2.3 Sampling and measurement

#### 2.3.1 Soybean plant sampling and analysis

Plant tissue sampling was performed at the soybean pod-filling stage (approximately 84 DAS, starting on September 7 annually) during both growing seasons. From each plot, three uniformly growing soybean plants with consistent vigor were selected at random to ensure sampling representativeness. The uppermost fully expanded trifoliate leaves (petioles completely removed) were collected as leaf samples, and intact fine roots (diameter < 2 mm, without root nodules) were excavated for root sampling. Unified sampling criteria were strictly enforced across all treatments and years to guarantee data reproducibility. All fresh plant samples were flash-frozen in liquid N_2_ and stored in a −80°C freezer for subsequent physiological and biochemical analyses. Fresh leaf and root samples were homogenized in pre-cooled phosphate buffer under ice-cold conditions and centrifuged at 4°C. The supernatant was collected as the crude enzyme extract. Peroxidase (POD) activity was determined spectrophotometrically by monitoring guaiacol oxidation in the presence of H_2_O_2_ at 470 nm (Scebba et al., 2001). Catalase (CAT) activity was determined by monitoring the decomposition of H_2_O_2_ at 240 nm (Cakmak and Marschner, 1992). Auxin (IAA) and cytokinin (CTK) were detected using high-performance liquid chromatography (Shimadzu LC-20AT, Japan) equipped with a C_18_ column (5 µm particle size) and a UV detector set at 254 nm (Zhou et al., 2025a). The mobile phase consisted of methanol and 0.6% (v/v) acetic acid (1:1, v/v), delivered at a flow rate of 1.0 mL min^-1^ and maintained at 35°C. Each sample was analyzed in triplicate to ensure reproducibility.

At physiological maturity, mature pods on main stems and branches were harvested per plot, and grain yield was measured after natural air drying to a constant weight. The aboveground plant residues were oven-dried and pulverized, then digested using the H_2_SO_4_-H_2_O_2_ method for elemental analysis. Total plant potassium concentration was determined via flame photometry, and total nitrogen concentration was measured using the distillation method. Aboveground nitrogen and potassium accumulation was calculated as the product of aboveground dry biomass and corresponding nutrient concentration, which was further adopted to evaluate plant nutrient uptake capacity.

#### 2.3.2 Soil sampling and physicochemical index determination

Topsoil samples were collected using a 5-cm-diameter soil corer from a depth of 0–20 cm at three random points in each experimental plot. Sampling was carried out during four key stages of soybean growth: the seedling stage, the flowering stage, the pod-filling stage, and the maturity stage. Soil AK was extracted with 1 mol L^−1^ CH_3_COONH_4_ (pH 7.0) at a soil-to-solution ratio of 1:10. After 30 min of continuous shaking and filtration, AK concentration was determined using a flame photometer (Lorenz et al., 2010). Soil IN was extracted using a 2 mol L^-1^ KCl solution and determined by a colorimetric method (Saha et al., 2018). During the pod-filling stage, additional soil samples were collected and divided into two parallel subsamples. One of the subsamples was used to determine soil enzyme activity, including protease, urease, acid phosphatase (ACP) and phosphodiesterase (PDE), whereas the other subsample was stored at –80°C for subsequent soil microbial DNA extraction and high-throughput sequencing analysis. Soil urease activity was determined by the colorimetric method using urea as substrate (Kandeler and Gerber, 1988); protease activity was measured with casein as substrate (Nannipieri et al., 1980); ACP and PDE activities were determined using p-nitrophenyl phosphate as substrate (Tabatabai and Bremner, 1969; Browman and Tabatabai, 1978).

### 2.4 Soil microbial community analysis

Soil samples were freeze-dried prior to microbial genomic DNA extraction. Total genomic DNA was isolated from soil samples using the D5625 Soil DNA Isolation Kit (Omega Bio-Tek, USA). The purity and integrity of the extracted DNA were validated through agarose gel electrophoresis and NanoDrop™ 2000 spectrophotometry, with key metrics including DNA concentration, structural integrity, and A260/A280 ratio. DNA samples that met the quality criteria were stored at −20°C for subsequent molecular analyses. High-throughput amplicon sequencing was employed to characterize the compositional profiles of soil bacterial and fungal communities. The V3–V4 hypervariable region of bacterial 16S rRNA genes was amplified using the primer pair 343F (5’-TACGGRAGGCAGCAG-3’) and 798R (5’-AGGGTATCTAATCCT-3’). The ITS1 region of fungal ITS rRNA genes was amplified using the primers ITS1F (5’-CTTGGTCATTTAGAGGAAGTAA-3’) and ITS2 (5’-GCTGCGTTCTTCATCGATGC-3’), following the experimental protocol of Mukherjee et al. (2014). All polymerase chain reaction (PCR) amplifications were conducted using Takara Ex Taq DNA polymerase with specifically optimized thermal cycling conditions. Following amplification, the resulting PCR products were purified and subjected to index PCR to enable sample tagging and multiplexing. The concentrations of constructed amplicon libraries were quantified using the Qubit dsDNA HS Assay Kit. Library sequencing was performed on the Illumina NovaSeq 6000 platform (Illumina Inc., USA) with a 2 × 250 bp paired-end read strategy, and all sequencing services were provided by OE Biotech Co., Ltd. (Shanghai, China).

Raw FASTQ sequencing data were processed using standardized bioinformatic pipelines. Briefly, Cutadapt software was used to trim adapter sequences from raw reads. Quality filtering, denoising, sequence merging, and chimera removal were performed using the DADA2 algorithm embedded in the QIIME2 (2020.11) platform with default parameters (Bolyen et al., 2019). The raw read count of individual samples ranged from 78,139 to 81,952, and clean tag numbers after quality control varied from 45,051 to 77,112. After chimera elimination, valid tags for subsequent analysis ranged from 42,311 to 76,208 per sample, which was sufficient to capture the full spectrum of soil microbial diversity (Li et al., 2026; Fu et al., 2022). The amplicon sequence variant (ASV) number per sample ranged from 171 to 1,775. Representative sequences and ASV abundance matrices were generated via QIIME2 analysis. Taxonomic annotation of representative ASVs was performed against the Silva 138 database using the q2-feature-classifier plugin with default settings. QIIME2 was used to calculate microbial alpha diversity indices (Chao1, Shannon, and Simpson) to evaluate community richness and diversity. The raw sequence data from this study can be found on the Sequence Read Archive of the National Centre for Biotechnology Information, with the BioProject number PRJNA1268030.

### 2.5 Calculations and statistical analysis

Soybean potassium use efficiency (KUE) and nitrogen use efficiency (NUE) were calculated using the following formulas:

KUE (%) = (plant K uptake in the K application treatment – plant K uptake in the NKF treatment)/K application amount × 100

NUE (%) = (plant N uptake in the N application treatment – plant N uptake in the NNF treatment)/N application amount × 100

Raw experimental data were sorted and preliminarily screened using Microsoft Excel 2019. Statistical analyses were performed with IBM SPSS Statistics v22.0. For each year, differences among fertilization treatments were analyzed by one-way ANOVA followed by Duncan’s multiple range test at *p* < 0.05. Two-way ANOVA was used to evaluate the effects of year, treatment, and their interaction. For the four K-fertilized treatments, three-way ANOVA was further performed to test the effects of year, fertilizer type, placement method, and their interactions. All data visualization was completed using Origin Pro 2021. For microbial data analysis, the ASV abundance matrix was normalized using the “decostand” function in the R vegan package (v4.2.0) to eliminate biases caused by uneven sequencing depth. Principal coordinate analysis (PCoA) based on Bray–Curtis distance matrices was conducted to characterize microbial beta diversity and community structural differences among treatments, and permutational multivariate analysis of variance (PERMANOVA) was used to test the significance of treatment effects. Microbial community abundance was visualized via the online Tutools platform. The linear discriminant analysis effect size (LEfSe) method was applied to identify different taxa within the soil microbial community among treatments, and taxa with *p* < 0.05 and an LDA score > 3.0 were regarded as discriminant genera. Microbial co-occurrence networks were constructed based on the top 100 most abundant bacterial and fungal genera. Pairwise Spearman correlation analysis was conducted between genera, and only robust and significant correlations (|r| > 0.6, *p* < 0.01) after false discovery rate correction were retained for network construction. R software (v4.4.2) with the “linkET”, “igraph”, “dplyr”, and “readxl” packages was used for correlation calculation and data sorting. The Gephi visualization tool was adopted to construct co-occurrence networks, divide network modules, and compute topological parameters. Structural equation modeling (SEM) was performed in R to clarify the causal connections between fertilizer type, fertilization mode, soil microbial diversity, soil enzyme activity, plant growth characteristics, soil fertility, and soybean productivity (Zhao et al., 2019). Model fitness was evaluated based on standard statistical criteria, with acceptable fitting defined as χ^2^/df ≤ 3, GFI ≥ 0.8, and RMSEA ≤ 0.08 (Guo et al., 2026; Cao et al., 2022).

## 3 Results

### 3.1 Soybean productivity

Soybean yield was significantly affected by different treatments, year, and their interaction (*p* < 0.01; **Table 1**). The interaction between fertilizer type and placement method also significantly affected soybean yield (*p* < 0.001; **Supplementary Table 1**). Compared with NKF, K fertilized treatments increased yield by 12.9%–22.9% in 2023 and 15.6%–31.6% in 2024. Across the two K fertilizer types, deep banding produced slightly higher soybean yield than broadcast incorporation, with increases of 1.1%–2.7% in 2023 and 3.7%–3.8% in 2024. The CPS formulation further intensified this effect, leading to 6.0%–7.7% higher yields in 2023 and 9.6%–9.7% higher in 2024 compared to PS (**Table 1**). Hierarchical evaluation of the treatments over the two years consistently ranked the yields as: DCPS > BCPS > DPS > BPS > NNF > NKF. The DCPS treatment consistently ranked first among the K-fertilized treatments, yielding 1.1%–8.9% higher in 2023 and 3.7%–13.8% higher in 2024 than the other K treatments, with the effect being more pronounced in the second year.

**Table 1:** Soybean yield, potassium use efficiency (KUE), and nitrogen use efficiency (NUE) in different treatments during the 2023 and 2024 soybean growing seasons.

Year	Treatment	Yield (kg ha^-1^)	KUE (%)	NUE (%)
2023	NNF	4560.2 ± 108.5c	27.1 ± 4.9d	–
	NKF	4268.8 ± 70.0d	–	29.5 ± 1.7c
	BPS	4820.4 ± 73.0b	32.4 ± 1.4cd	43.1 ± 1.6b
	DPS	4951.0 ± 56.9b	35.7 ± 0.2c	45.8 ± 6.2b
	BCPS	5189.8 ± 50.8a	42.6 ± 5.7b	54.0 ± 2.8a
	DCPS	5248.2 ± 70.0a	49.9 ± 3.2a	56.8 ± 0.4a
2024	NNF	4496.9 ± 28.5e	29.4 ± 5.6c	–
	NKF	4204.6 ± 107.8f	–	29.9 ± 1.2c
	BPS	4862.6 ± 56.1d	38.3 ± 2.4b	44.5 ± 1.6b
	DPS	5045.7 ± 66.9c	39.8 ± 5.9b	47.0 ± 5.6b
	BCPS	5331.2 ± 98.2b	47.9 ± 3.3a	58.7 ± 2.2a
	DCPS	5531.8 ± 77.4a	57.2 ± 2.6a	62.6 ± 5.3a
*p* value				
	Year (Y)	0.008**	0.034*	0.409 NS
	Treatment (T)	0.000***	0.000***	0.018*
	Y×T	0.004**	0.980 NS	0.981 NS

Values are means ± standard deviation (SD, n = 3). Different lowercase letters indicate significant differences among treatments within the same year and column at *p* < 0.05. “–” indicates not applicable. Asterisks ‘*’, ‘**’, and ‘***’ represent significance at 0.05, 0.01, and 0.001 probability level, respectively, and ‘NS’ denotes non-significance.

KUE and NUE differed significantly among treatments (*p* < 0.05), whereas the interaction between year and treatment was not significant (**Table 1**). The fertilizer type × placement method interaction was significant for KUE (*p* < 0.05) but not for NUE (**Supplementary Table 1**). Both the CPS application and deep banding of K fertilizer resulted in high KUE and NUE. Compared to the PS treatments, the KUE and NUE in the CPS treatments were higher by 10.2–14.2 and 10.9–11.0 percentage points in 2023, and 9.6–14.4 and 14.2–15.6 percentage points in 2024, respectively. Deep banding increased KUE and NUE relative to broadcast incorporation by 3.3–7.3 and 2.7–2.8 percentage points in 2023, and 1.5–9.3 and 2.5–3.9 percentage points in 2024, respectively. Among treatments with calculable values, DCPS showed the highest KUE and NUE, surpassing the other treatments by 7.3–22.8 and 2.8–27.3 percentage points in 2023, and by 9.3–27.8 and 3.9–32.7 percentage points in 2024, respectively.

### 3.2 Plant physiological traits of soybean

**Figure 1:**
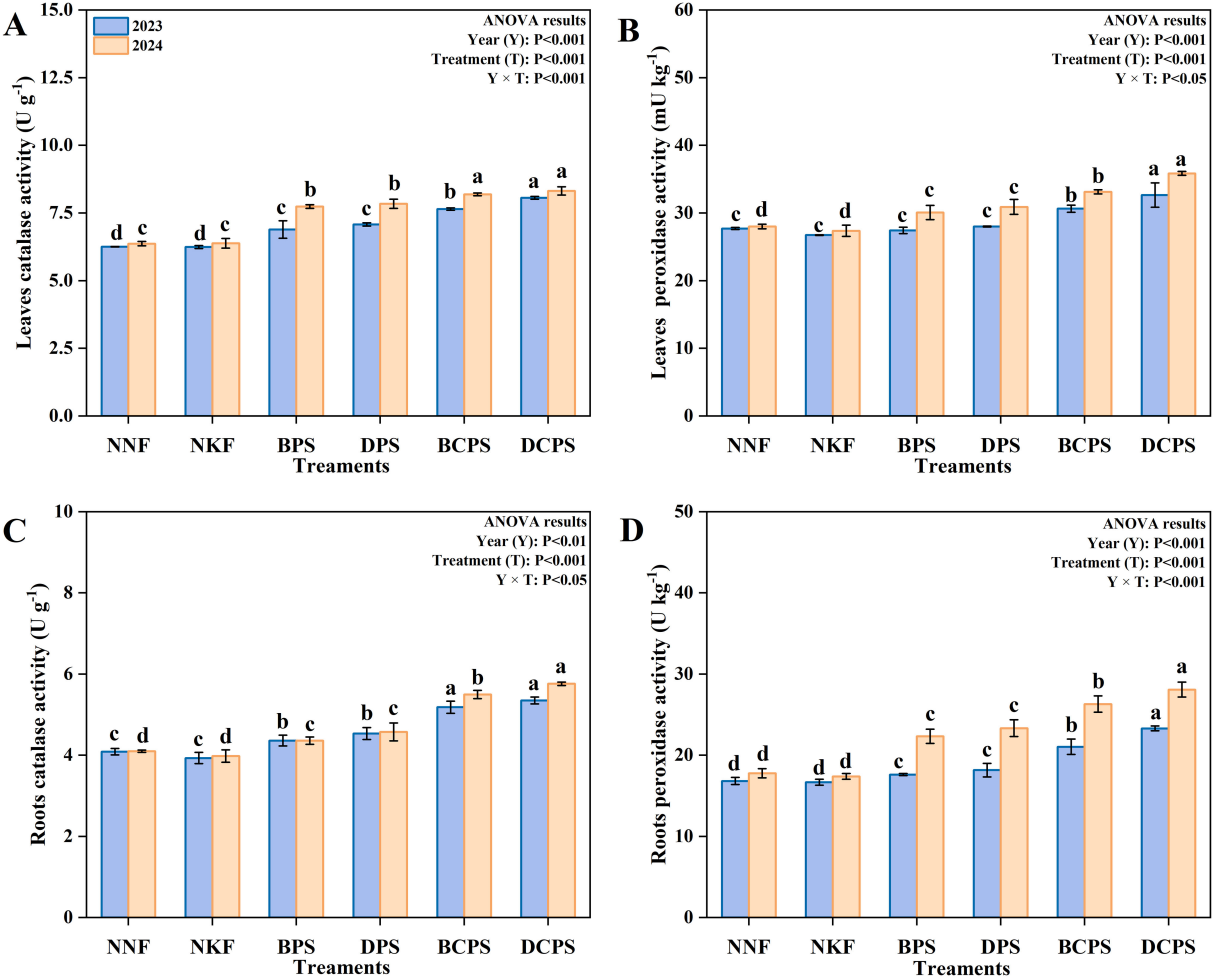
Catalase and peroxidase activities in soybean leaves and roots in different treatments during the 2023 and 2024 growing seasons. **(A)** Leaves catalase activity; **(B)** Leaves peroxidase activity; **(C)** Roots catalase activity; **(D)** Roots peroxidase activity. Error bars represent SD values; Different lowercase letters represent significant differences at *p* < 0.05. NNF, NKF, BPS, DPS, BCPS, and DCPS indicate nitrogen-free fertilization, potassium-free fertilization, PS with broadcast incorporation, PS with deep banding, CPS with broadcast incorporation, and CPS with deep banding, respectively.

Both CAT and POD activities in soybean leaves and roots were significantly affected by treatment, year, and their interaction (*p* < 0.05; **Figure 1**), as well as by the interaction between fertilizer type and placement method (*p* < 0.05; **Supplementary Table 1**). In 2023, all K-supplemented treatments significantly elevated leaf CAT and POD activities by 10.4%–29.2% and 2.6%–22.2%, and root CAT and POD activities by 11.0%–36.1% and 5.6%–39.7% compared to the NKF control. Such stimulatory effects were further pronounced in 2024, with corresponding increases of 21.3%–30.4% (leaf CAT), 9.9%–31.0% (leaf POD), 9.5%–44.7% (root CAT), and 28.4%–61.5% (root POD) (**Figure 1**). Among all K fertilization regimes, DCPS treatment exerted the most prominent enhancement on soybean antioxidant enzyme activities, consistently outperforming BCPS and DPS across both experimental years. In 2023, DCPS increased leaf CAT activity by 5.4% and 13.9%, root CAT activity by 3.2% and 18.0%, leaf POD activity by 6.6% and 16.6%, and root POD activity by 10.7% and 28.3% compared with BCPS and DPS, respectively. In 2024, the corresponding increases were 1.6% and 6.1% for leaf CAT, 4.8% and 25.9% for root CAT, 8.2% and 16.0% for leaf POD, and 6.8% and 24.0% for root POD (**Figure 1**).

**Figure 2:**
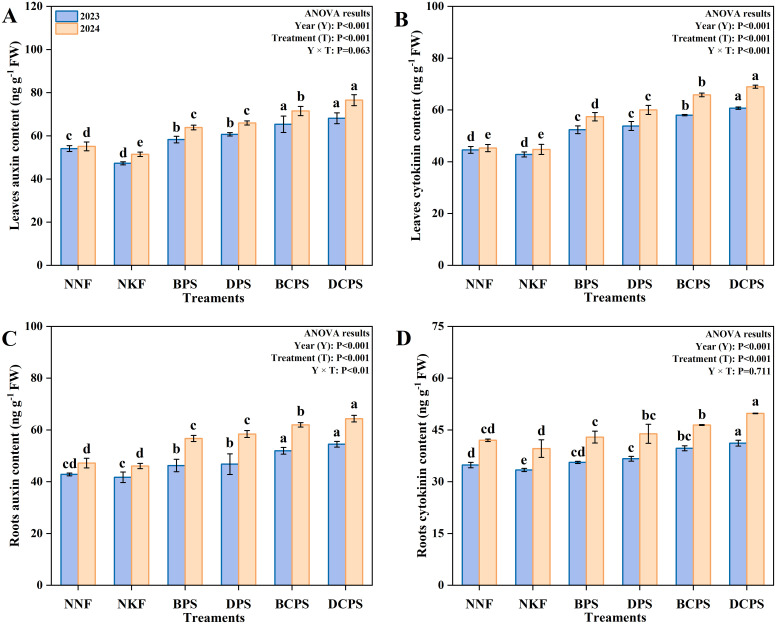
Auxin and cytokinin contents in soybean leaves and roots in different treatments during the 2023 and 2024 growing seasons. **(A)** Leaves auxin content; **(B)** Leaves cytokinin content; **(C)** Roots auxin content; **(D)** Roots cytokinin content. Error bars represent SD values; Different lowercase letters represent significant differences at *p* < 0.05. NNF, NKF, BPS, DPS, BCPS, and DCPS indicate nitrogen-free fertilization, potassium-free fertilization, PS with broadcast incorporation, PS with deep banding, CPS with broadcast incorporation, and CPS with deep banding, respectively.

Similarly, fertilization treatment and year significantly affected IAA and CTK contents in soybean leaves and roots (*p* < 0.001), whereas the interaction between year and treatment was significant only for leaf CTK and root IAA contents (*p* < 0.01; **Figure 2**). The interaction between fertilizer type and placement method also significantly affected IAA and CTK contents in soybean leaves and roots (*p* < 0.01; **Supplementary Table 1**). The DCPS treatment maintained the highest IAA and CTK concentrations in both aboveground and underground plant tissues throughout the trial period. Specifically, DCPS increased leaf IAA content by 4.2%–44.1% (2023) and 7.1%–48.9% (2024), and leaf CTK content by 4.7%–41.8% (2023) and 4.8%–54.1% (2024). For root tissues, IAA content was elevated by 4.9%–30.5% in 2023 and 3.9%–39.7% in 2024, while CTK content increased by 3.7%–23.3% (2023) and 7.2%–25.7% (2024) under DCPS fertilization (**Figure 2**). Collectively, these findings verify that the integration of controlled-release potassium sulfate fertilizer and deep banding cultivation generates positive synergistic effects. This optimized fertilization strategy effectively modulates plant antioxidant defense systems and endogenous hormone homeostasis, thereby providing a favorable physiological foundation for sustainable soybean growth and development.

### 3.3 Soil physicochemical properties and enzyme activities

Fertilization treatment and year had significant effects on soil IN and AK contents (*p* < 0.05), whereas their interaction was significant only for soil AK (**Table 2**). The interaction between fertilizer type and placement method also significantly influenced both IN and AK contents (*p* < 0.05; **Supplementary Table 1**). All K-fertilized treatments produced markedly higher soil AK concentrations than the NKF control, with the DCPS treatment recording the greatest AK accumulation (*P* < 0.05). In 2023, DCPS increased soil AK content by 7.4%, 6.3%, and 2.2% relative to BPS, DPS, and BCPS, respectively, while comparable increases of 7.0%, 3.2%, and 2.1% were observed in 2024. Soil IN content was substantially reduced under N deficiency, as evidenced by the lower IN values in the NNF treatment. Relative to NKF, K fertilization improved soil IN levels by 6.4%–11.0% in 2023 and 2.5%–8.0% in 2024. Compared with conventional PS fertilization, CPS application increased soil IN content by 2.4%–2.5% in 2023 and 2.8%–3.7% in 2024. Additionally, deep banding consistently outperformed broadcast incorporation in elevating soil IN availability, with increases of 1.7%–1.8% in 2023 and 1.6%–2.5% in 2024.

**Table 2:** Mean values of soil inorganic N (IN) and available K (AK) in different treatments during the 2023 and 2024 soybean growing seasons.

Year	Treatment	IN (mg kg^−1^)	AK (mg kg^−1^)
2023	NNF	25.3 ± 0.6d	123.8 ± 4.7b
	NKF	26.4 ± 1.5c	107.0 ± 1.7c
	BPS	28.1 ± 0.5b	124.0 ± 1.7b
	DPS	28.6 ± 0.1ab	125.3 ± 1.4b
	BCPS	28.8 ± 1.1ab	130.3 ± 1.5a
	DCPS	29.3 ± 0.4a	133.2 ± 2.1a
2024	NNF	21.8 ± 0.7d	125.9 ± 1.6bc
	NKF	23.9 ± 0.4c	99.2 ± 3.5d
	BPS	24.5 ± 0.3bc	124.1 ± 4.5c
	DPS	25.1 ± 0.5ab	128.7 ± 0.5ab
	BCPS	25.4 ± 1.3ab	130.1 ± 1.7a
	DCPS	25.8 ± 0.5a	132.8 ± 1.5a
*p* value			
	Year (Y)	0.000***	0.600 NS
	Treatment (T)	0.000***	0.000***
	Y×T	0.222 NS	0.016*

Values are means ± standard deviation (SD, n = 3). Different lowercase letters indicate significant differences among treatments within the same year and column at *p* < 0.05. “–” indicates not applicable. Asterisks ‘*’, ‘**’, and ‘***’ represent significance at 0.05, 0.01, and 0.001 probability level, respectively, and ‘NS’ denotes non-significance.

**Figure 3:**
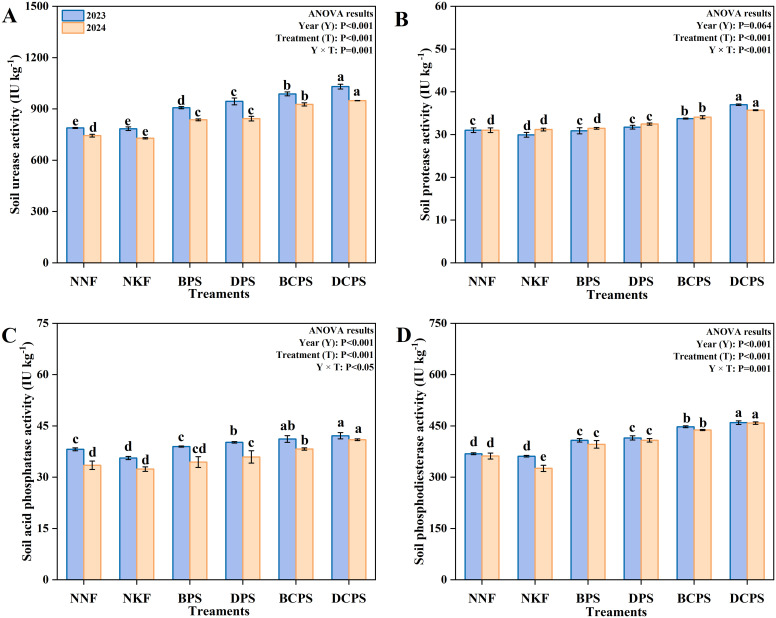
Soil enzyme activities in different K fertilization treatments during the 2023 and 2024 soybean growing seasons. **(A)** Soil urease activity; **(B)** Soil protease activity; **(C)** Soil acid phosphatase activity; **(D)** Soil phosphodiesterase activity. Error bars represent SD values; Different lowercase letters represent significant differences at *p* < 0.05. NNF, NKF, BPS, DPS, BCPS, and DCPS indicate nitrogen-free fertilization, potassium-free fertilization, PS with broadcast incorporation, PS with deep banding, CPS with broadcast incorporation, and CPS with deep banding, respectively.

Fertilization treatment significantly influenced all soil enzyme activities, whereas year significantly affected urease, acid phosphatase, and phosphodiesterase activities (*p* < 0.001; **Figure 3**). The interactions between treatment and year as well as between fertilizer type and placement method also significantly affected soil enzyme activities (*p* < 0.05; **Supplementary Table 1**). The DCPS treatment substantially enhanced the activities of nitrogen-cycle enzymes (protease and urease) and phosphorus-cycle enzymes (ACP and PDE) in both growing seasons, exhibiting stronger stimulatory effects than all alternative fertilization regimes. In 2023, DCPS increased protease, urease, ACP, and PDE activities by 9.6%–23.5%, 4.3%–31.4%, 2.3%–18.3%, and 2.7%–27.1%, respectively. In 2024, the corresponding elevation ranges were 4.8%–15.1%, 2.4%–30.2%, 7.1%–26.5%, and 4.6%–40.6%, demonstrating consistent and robust improvements in soil biochemical function under DCPS treatment.

### 3.4 Composition and diversity of soil microbial communities

#### 3.4.1 Diversity and community structure of soil bacteria and fungi

**Figure 4:**
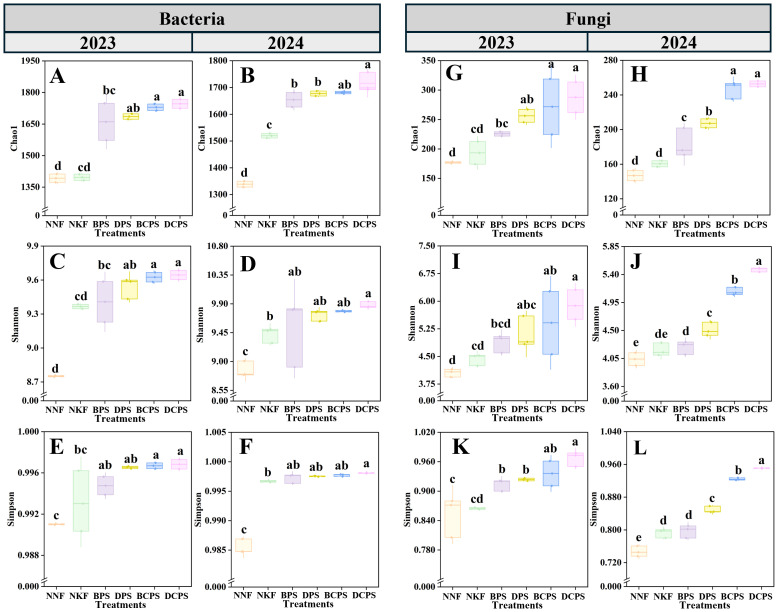
Alpha diversity (Chao1, Shannon, and Simpson indices) of soil bacterial **(A–F)** and fungal **(G–L)** communities in different treatments in 2023 and 2024. Different letters above the boxes indicate significant differences between treatments (ANOVA test, *p* < 0.05).

Soil microbial alpha diversity, evaluated via Chao1, Shannon, and Simpson indices at the soybean pod-filling stage, was significantly altered by different fertilization strategies in both experimental years (**Figure 4**). Both NNF and NKF treatments significantly reduced the diversity and richness of soil fungal and bacterial communities. Across K fertilization practices, deep banding generally increased fungal and bacterial alpha diversity indices relative to broadcast application, though these differences were not statistically significant (*p* > 0.05). Compared with conventional PS, CPS application markedly improved fungal and bacterial community diversity (*p* < 0.05), with the DCPS treatment achieving the optimal microbial diversity outcomes. Quantitative analysis revealed that DCPS increased bacterial Chao1, Shannon, and Simpson indices by 1.0%–25.4%, 0.2%–10.2%, and 0.1%–0.6% in 2023, and by 2.1%–28.2%, 1.0%–11.4%, and 0.1%–1.3% in 2024. For fungal communities, DCPS produced more pronounced improvements, with Chao1, Shannon, and Simpson indices elevated by 5.9%–62.6%, 9.0%–45.3%, and 3.3%–13.5% in 2023, and by 2.5%–72.1%, 6.9%–35.7%, and 2.9%–27.2% in 2024.

**Figure 5:**
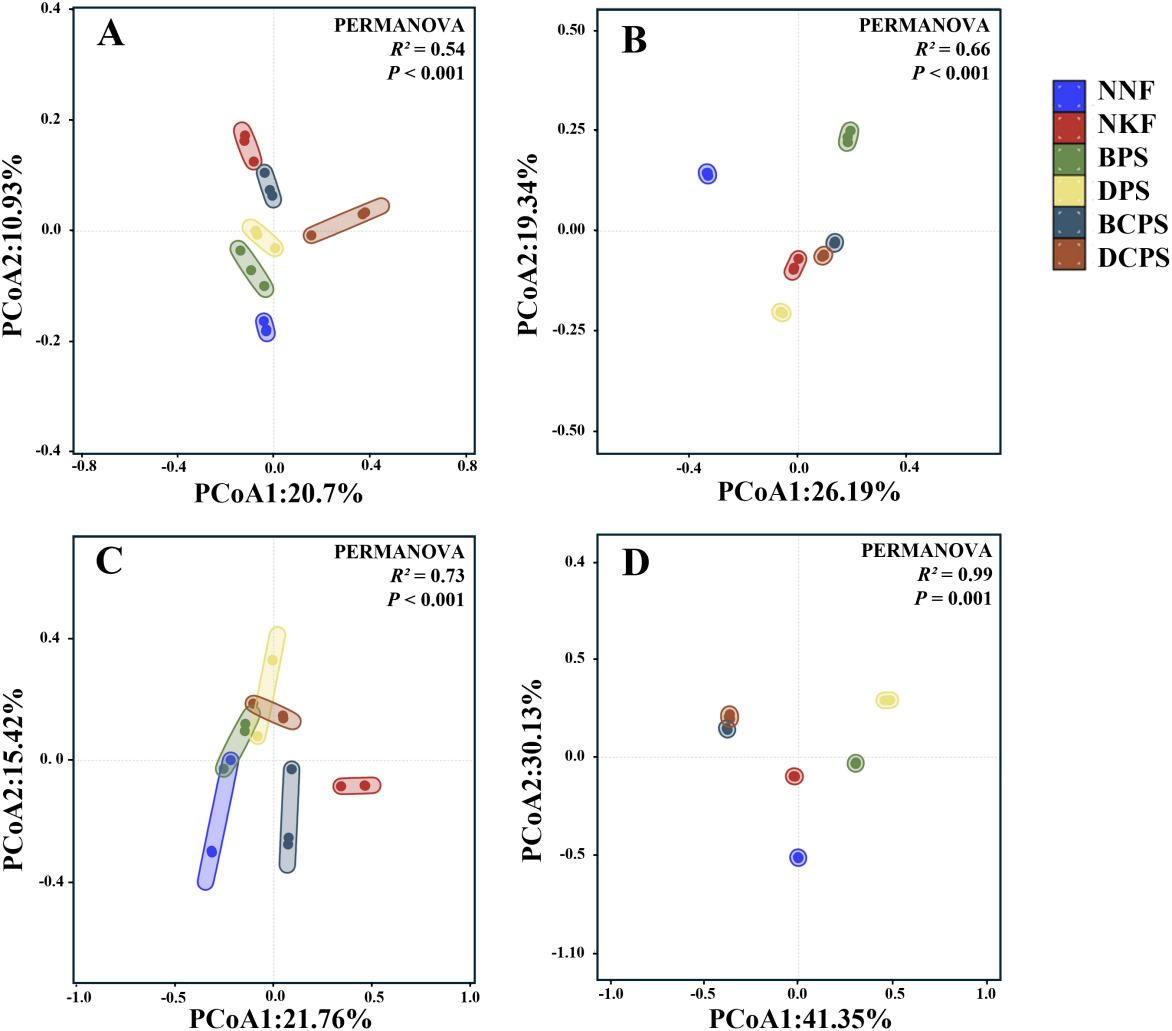
Principal Coordinates Analysis (PCoA) of the effects of different treatments on soil bacterial and fungal communities in 2023 and 2024. **(A–D)** 2023 bacterial, 2024 bacterial, 2023 fungal, and 2024 fungal, respectively. R² and p values from PERMANOVA indicate the explained variation and significance of treatment effects, respectively.

Principal coordinate analysis (PCoA) was performed to characterize variations in microbial community structure across treatments (**Figure 5**). PERMANOVA further showed significant differences in bacterial and fungal community composition among treatments in both 2023 and 2024 (*p* < 0.001). For bacterial communities, PCoA1 explained 20.7% and 26.2% of total variation in 2023 and 2024, while PCoA2 accounted for 10.9% and 19.3%, respectively. For fungal communities, PCoA1 contributed 21.8% (2023) and 41.4% (2024) of the structural variance, and PCoA2 explained 15.4% (2023) and 30.1% (2024). The PCoA ordinations clearly demonstrated that soil microbial community structure under DCPS was distinctly separated from that of all other treatments, indicating that CPS deep banding induced unique structural shifts in soil bacterial and fungal assemblages.

#### 3.4.2 The composition of soil bacteria and fungi communities

**Figure 6:**
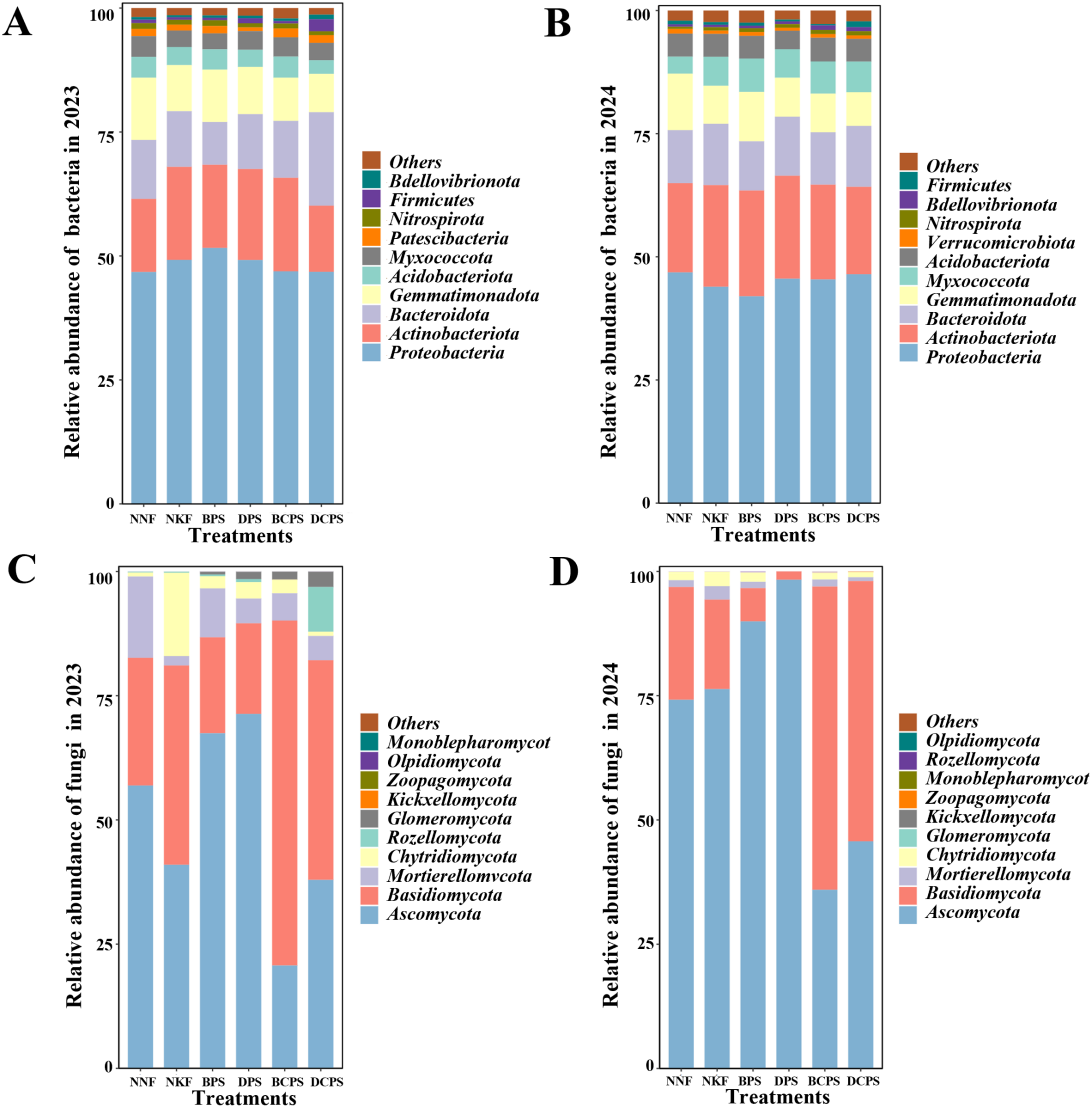
Relative abundances of dominant bacterial and fungal phyla in different fertilization treatments. **(A)** Bacterial in 2023; **(B)** bacterial in 2024; **(C)** fungal in 2023; and **(D)** fungal in 2024.

Across both experimental years, the soil bacterial community was predominantly composed of six core phyla, including *Actinobacteriota*, *Proteobacteria*, *Gemmatimonadota*, *Bacteroidota*, *Myxococcota*, and *Acidobacteriota*. Collectively, these phyla accounted for 92.5%–96.3% of the total bacterial sequences across all treatments (**Figures 6A, B**), indicating that different fertilization regimes did not alter the fundamental taxonomic framework of soil bacterial communities. Similarly, the fungal community was primarily composed of *Mortierellomycota*, *Basidiomycota*, *Ascomycota*, and *Chytridiomycota*, which collectively occupied 82.0%–99.9% of total fungal reads (**Figures 6C, D**).

**Figure 7:**
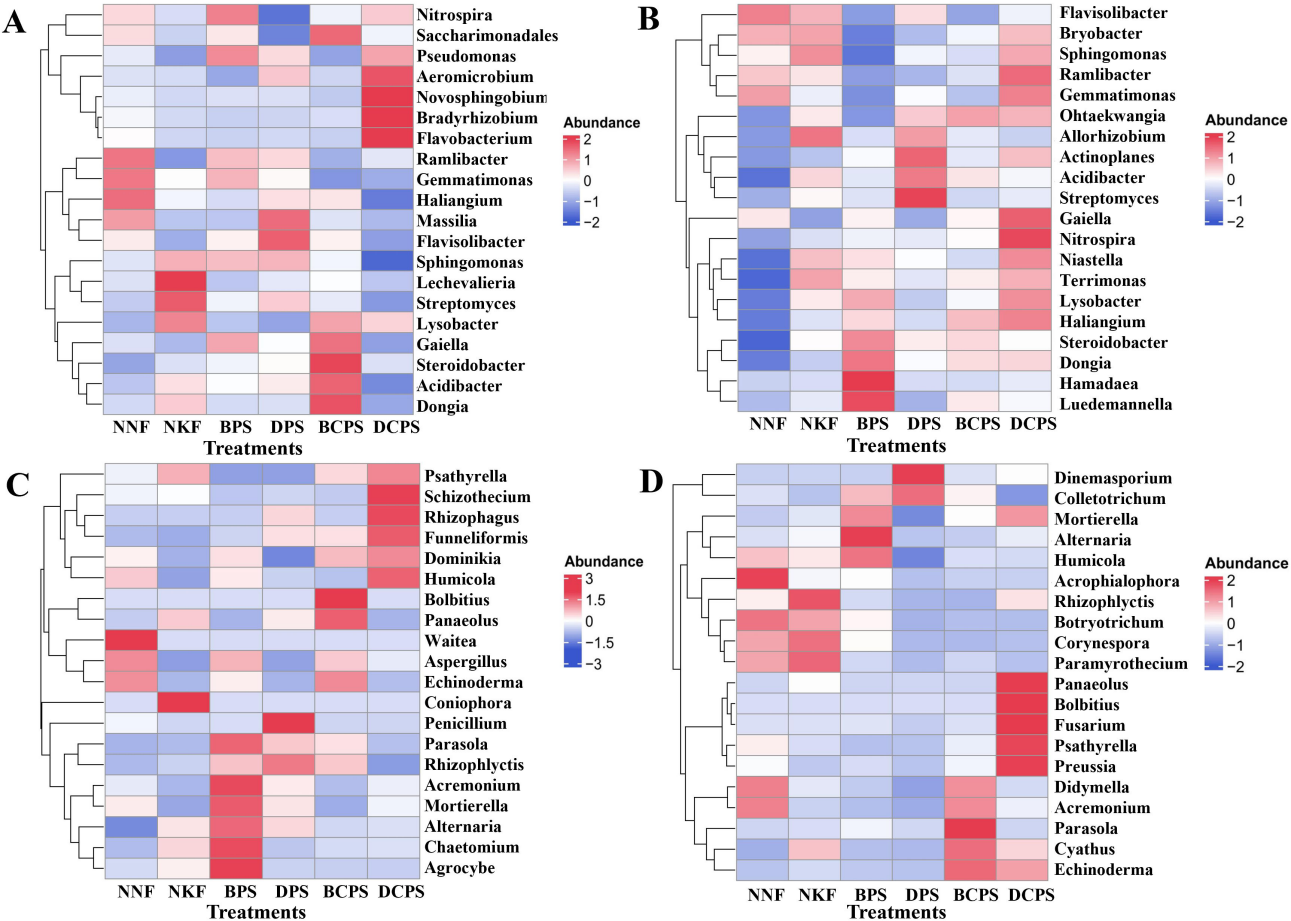
Heatmaps of bacterial [**(A)** for 2023, **(B)** for 2024] and fungal [**(C)** for 2023, **(D)** for 2024] communities at the genus level in the different K fertilization treatments.

Genus-level analysis of the top 20 dominant microbial taxa revealed pronounced treatment-specific enrichment patterns under DCPS (**Figure 7**). In 2023, DCPS significantly enriched the relative abundances of *Aeromicrobium*, *Novosphingobium*, *Bradyrhizobium*, and *Flavobacterium* compared with other treatments. In 2024, DCPS significantly increased the relative abundances of *Ramlibacter*, *Gemmatimonas*, *Gaiella*, *Nitrospira*, and *Niastella*. Cluster analysis of fungal genera revealed treatment-specific community responses (**Figures 7C, D**). In 2023, DCPS markedly elevated the relative abundances of *Schizothecium*, *Rhizophagus*, *Funneliformis*, *Dominikia*, and *Humicola*. In 2024, the genera *Panaeolus*, *Bolbitius*, *Fusarium*, and *Preussia* were significantly enriched under DCPS. Notably, the fungal genus *Psathyrella* consistently maintained a higher relative abundance in the DCPS treatment across both years.

**Figure 8:**
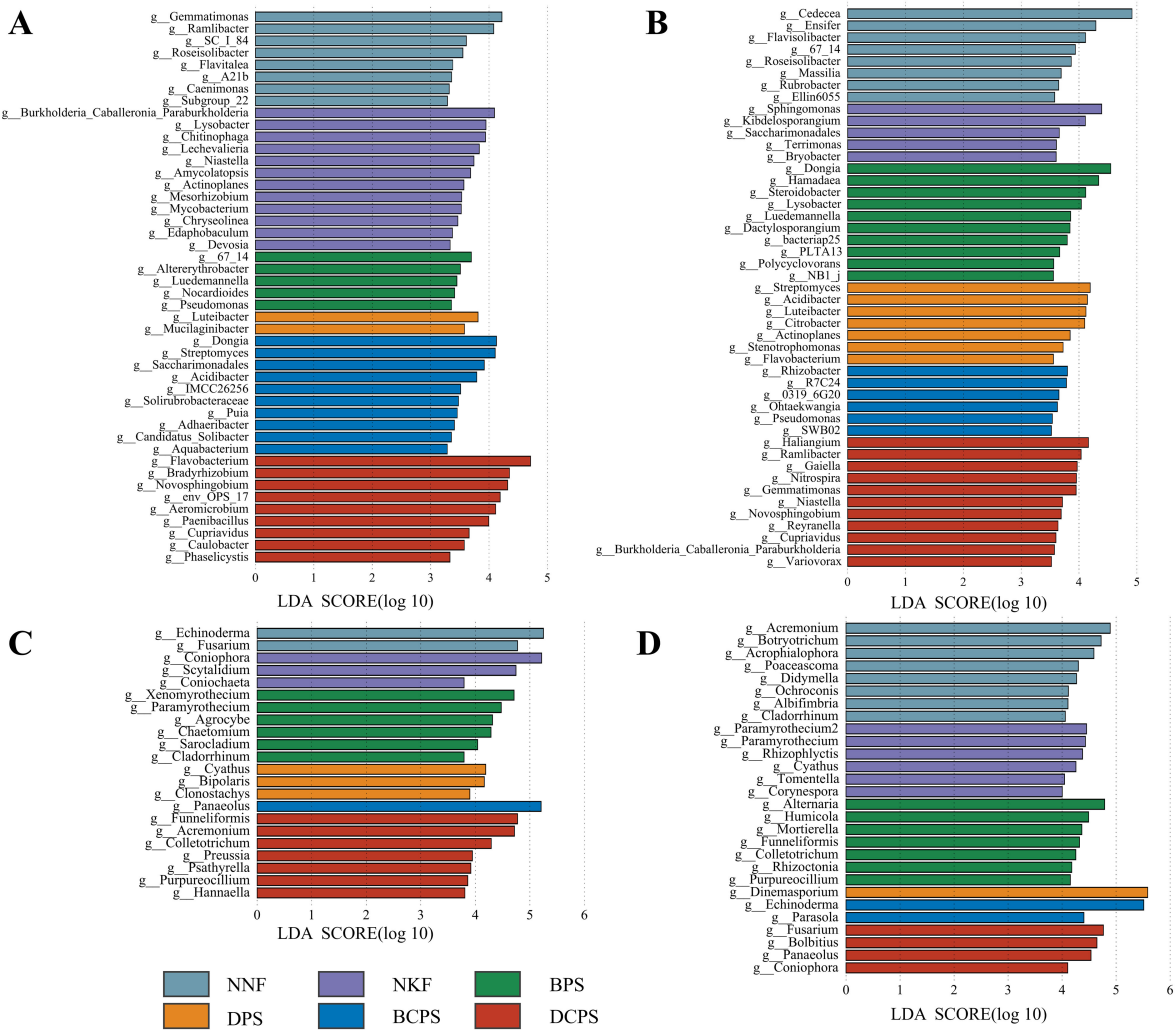
Linear discriminant analysis effect size (LEfSe) of soil microbial communities. **(A)** Bacterial community in 2023; **(B)** bacterial community in 2024; **(C)** fungal community in 2023; **(D)** fungal community in 2024. Differentially abundant taxa were identified using the Kruskal–Wallis test (*p* ≤ 0.05), and their effect sizes were estimated by linear discriminant analysis (LDA).

LEfSe analysis further identified discriminant genera that responded to the integrated CPS and deep banding treatment (**Figure 8**). In bacterial communities, DCPS was characterized by higher LDA scores for *Flavobacterium*, *Bradyrhizobium*, *Novosphingobium*, and *Aeromicrobium* in 2023, and for *Haliangium*, *Ramlibacter*, *Gaiella*, *Nitrospira*, and *Gemmatimonas* in 2024. In fungal communities, *Funneliformis*, *Acremonium*, *Colletotrichum*, *Psathyrella*, and *Purpureocillium* were discriminant genera for DCPS in 2023, whereas *Fusarium*, *Bolbitius*, and *Panaeolus* were enriched under DCPS in 2024.

#### 3.4.3 Structural characteristics of microbial co-occurrence networks

**Figure 9:**
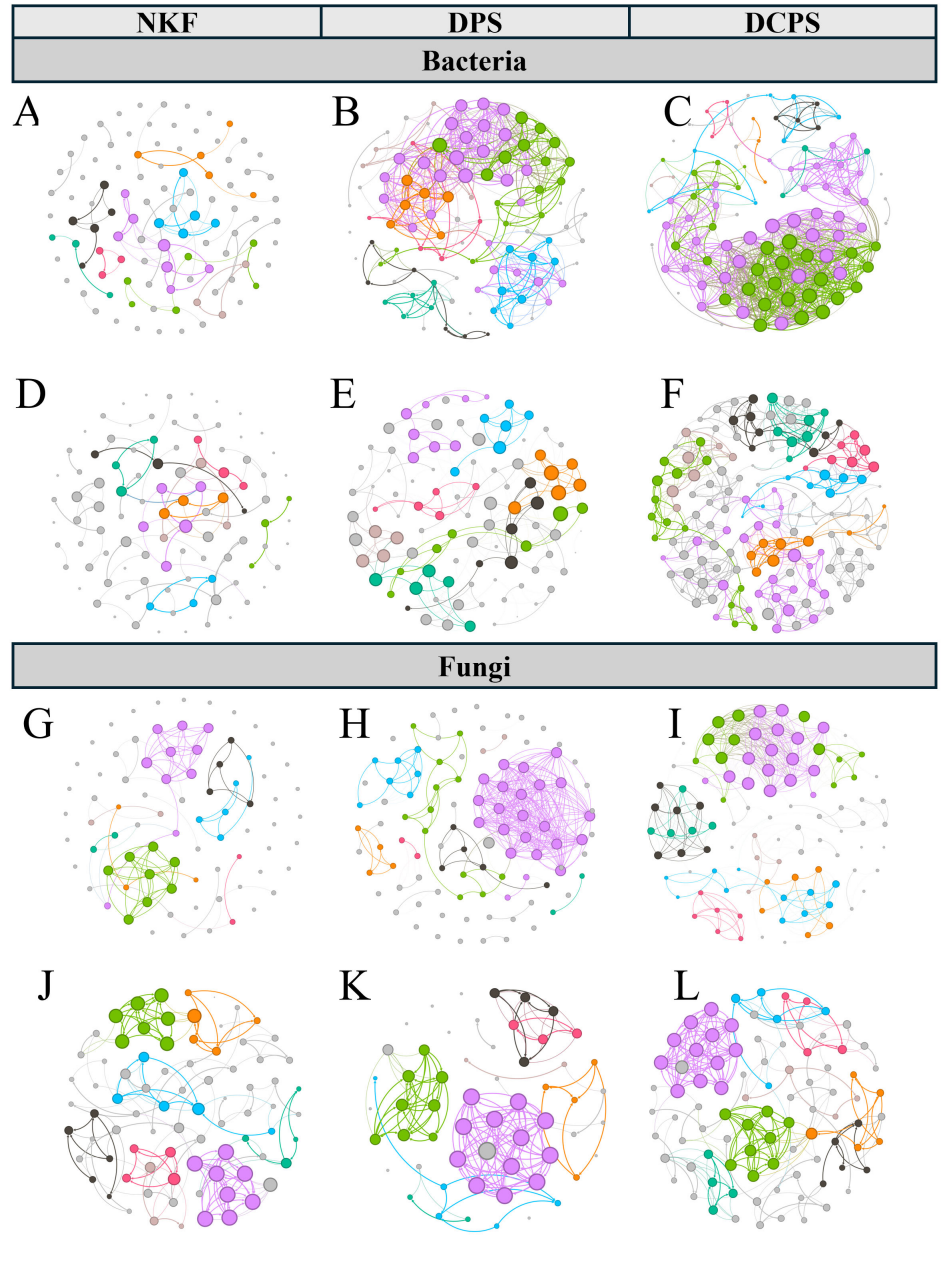
Co-occurrence networks of bacterial [**(A–C)** for 2023, **(D–F)** for 2024] and fungal [**(G–I)** for 2023, **(J–L)** for 2024] communities across sampling years.

Microbial co-occurrence network analysis revealed that DCPS generally promoted the formation of more complex and connected soil microbial networks during the two-year field experiment (**Figure 9**). The DCPS networks consistently had more edges than the DPS and NKF networks in both bacterial and fungal communities, with 762 and 508 bacterial edges and 433 and 240 fungal edges in 2023 and 2024, respectively (**Supplementary Table 2**). In bacterial networks, the average degree under NKF, DPS, and DCPS was 1.3, 9.6, and 15.2 in 2023; 1.4, 4.7, and 10.2 in 2024, respectively. In fungal networks, the average degrees were 2.3, 6.2, and 8.8 in 2023; 4.1, 5.3, and 5.4 in 2024, respectively. Consistent with the enhanced network connectivity, both bacterial and fungal networks exhibited the highest average clustering coefficients across the two years. The two-year mean average clustering coefficient under DCPS was 11.8% and 3.1% higher than NKF and DPS in bacterial networks, and 14.0% and 8.0% higher in fungal networks, respectively.

### 3.5 Correlation analysis of microbial community and environmental parameters

**Figure 10:**
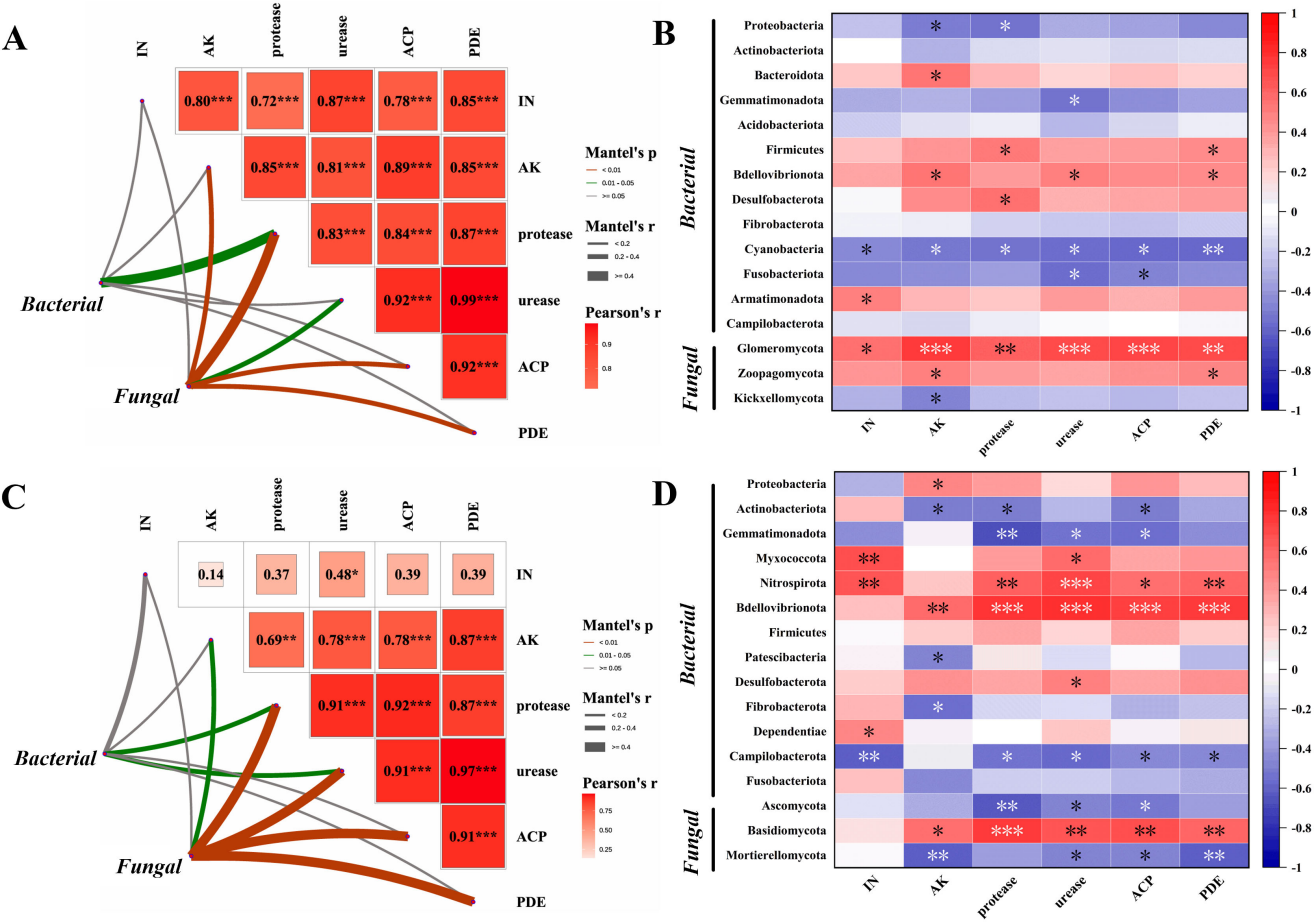
Mantel test between soil bacterial and fungal phyla and environmental factors in 2023 **(A)** and 2024 **(C)**. IN, inorganic nitrogen; AK, available potassium; protease; urease; ACP, acid phosphatase; PDE, phosphodiesterase. Spearman’s correlations between the relative abundances of soil bacterial and fungal phyla and environmental factors in 2023 **(B)** and 2024 **(D)**. The environmental factors include soil chemical properties and enzyme activities. *, **, and *** represent significant correlations at the 0.05, 0.01, and 0.001 levels.

Mantel test results showed significant correlations between soil bacterial community composition (phylum level) and multiple soil environmental parameters. Specifically, bacterial community structure was significantly correlated with soil protease activity in both years and urease activity in 2024 (**Figures 10A, C**). Fungal community composition exhibited broader environmental correlations, with significant associations with soil AK content and the activities of protease, urease, ACP, and PDE across both growing seasons.

Spearman correlation analysis further identified taxon-specific relationships between microbial communities and soil biochemical properties (**Figures 10B, D**). In 2023, the phylum *Glomeromycota* was positively correlated with soil IN, AK, protease, urease, ACP, and PDE. Firmicutes and *Bdellovibrionota* showed positive correlations with multiple soil enzyme indicators. By contrast, *Cyanobacteria* were negatively correlated with all measured soil properties, while *Gemmatimonadota*, *Fusobacteriota*, and *Kickxellomycota* exhibited negative correlations with specific soil nutrient and enzyme indices. In 2024, *Nitrospirota* and *Bdellovibrionota* were positively associated with soil AK and enzyme activities, whereas *Gemmatimonadota* and *Campilobacterota* exhibited negative correlations with key soil fertility indicators. For fungal phyla, *Basidiomycota* abundance was positively linked to AK concentration and ACP activity, while *Mortierellomycota* showed negative correlations with AK, urease, ACP, and PDE activities.

### 3.6 Driving mechanisms of fertilization regimes on soybean productivity

**Figure 11:**
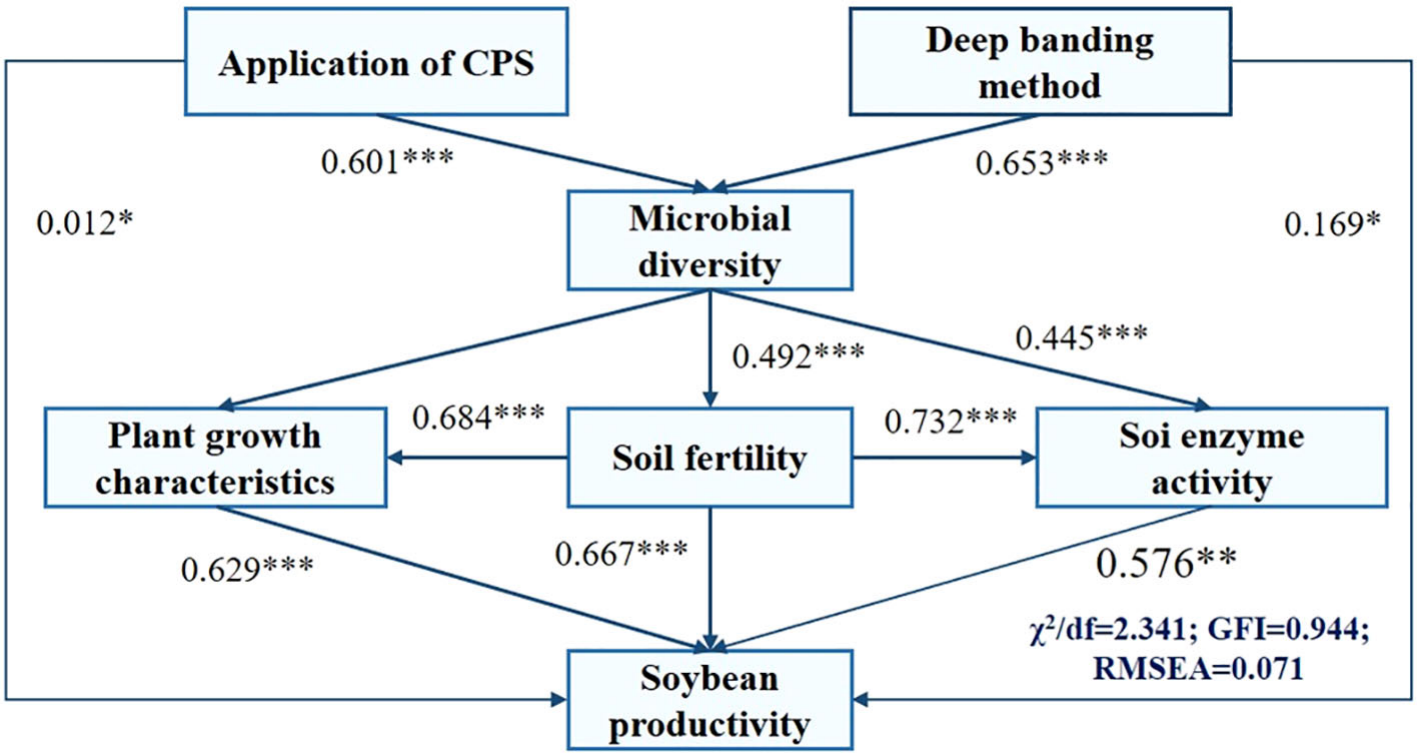
Structural equation model illustrating the effects of the deep banding method and application of CPS on soil properties and soybean productivity (χ^2^/df = 2.341, GFI = 0.944, RMSEA = 0.071). Path coefficients (correlation coefficients) beside arrows are standardized by the parameter means. Arrow width is proportional to the coefficient of the relationship (*, *p* < 0.05; **, *p* < 0.01; ***, *p* < 0.001). Each mediation variable is a collection of variables, i.e., microbial diversity includes bacterial and fungal alpha diversity, soil enzyme activities include protease, urease, acid phosphatase, and phosphodiesterase, soil fertility includes soil inorganic nitrogen and available potassium content, plant growth characteristics include antioxidant enzyme activities and endogenous hormone content, soybean productivity includes soybean yields and nitrogen and potassium use efficiency.

The SEM was used to clarify the causal pathways through which CPS fertilizer type and deep banding mode regulate soil fertility, microbial diversity, enzyme activity, plant physiological traits, and final soybean productivity (**Figure 11**). The established SEM model satisfied standard fitness criteria (χ^2^/df = 2.341, GFI = 0.944, RMSEA = 0.071). The modeling results confirmed that both CPS application and deep banding exerted significant direct positive effects on soybean yield performance (*p* < 0.05). Mechanistically, these two optimized fertilization practices significantly improved soil enzyme activity, soil fertility status, and plant growth attributes by modulating soil microbial diversity (*P* < 0.001). Soil microbial diversity served as a central upstream regulator, directly promoting soil enzyme activity, soil fertility, and plant physiological performance (*p* < 0.001, *p* < 0.001, *p* < 0.05, respectively). Improved soil fertility further enhanced soil enzyme activities and plant growth traits (*p* < 0.001). Ultimately, elevated soil enzyme activity, improved soil fertility, and optimized plant physiological characteristics collectively and significantly promoted soybean yield formation (*p* < 0.01, *p* < 0.001, *p* < 0.001, respectively). In summary, the integration of CPS and deep banding fertilization generated robust synergistic promotional effects. This optimized fertilization regime improved soybean productivity via a comprehensive cascade of ecological and physiological improvements, including enhanced soil microbial diversity, intensified nutrient-cycling enzyme activities, improved soil fertility, and optimized plant growth and physiological status.

## 4 Discussion

### 4.1 The impact on soybean productivity of different K fertilization schemes

As an indispensable macronutrient for crop growth, K significantly influences the agronomic performance of crops through its regulatory role in physiological processes (Ortel et al., 2025). However, intensive agricultural production often relies on excessive K fertilization to pursue high grain yields, which inevitably causes substantial soil K fixation and nutrient accumulation (Popp et al., 2020). Long-term irrational K input further triggers soil secondary salinization, disrupts soil nutrient balance, and deteriorates soil quality, ultimately impairing the stability and sustainability of crop yield production (Liu et al., 2021).

Optimized fertilization practices, such as deep banding, can enhance the agronomic performance of crops. Deep banding has been shown to enhance wheat grain yield (17.0%–19.7%), nutrient uptake efficiency (9.6%–25.2%), and nutrient use efficiency (80.1%–209.5%) compared to broadcasting (Liu et al., 2022). Moreover, controlled-release potassium fertilizers can synchronize nutrient release dynamics with crop seasonal nutrient demand, effectively improving crop yield and KUE compared with traditional K fertilizers (Qu et al., 2024). Consistent with previous studies, the present two-year field experiment confirmed the independent and synergistic advantages of CPS application and deep banding fertilization in improving soybean productivity and nutrient use efficiency. The DCPS treatment exhibited the optimal agronomic performance among all six fertilization regimes. The non-significant year × fertilizer type × placement method interaction for most indicators suggested that the effects of CPS and deep banding were generally consistent across years. Specifically, DCPS increased soybean yield by 15.1%, 22.9%, 8.9%, 6.0%, and 1.1% in 2023, and by 23.0%, 31.6%, 13.8%, 9.6%, and 3.8% in 2024, compared with NNF, NKF, BPS, DPS, and BCPS treatments, respectively (**Table 1**). In terms of nutrient utilization, DCPS also yielded superior nitrogen use efficiency (NUE) and KUE across both growing seasons. Relative to BPS, DPS, and BCPS, DCPS increased KUE by 17.5, 14.2, and 7.3 percentage points and NUE by 13.7, 11.0, and 2.8 percentage points in 2023, with further increases of 18.9, 17.4, 9.3 percentage points (KUE) and 18.1, 15.6, 3.9 percentage points (NUE) observed in 2024 (**Table 1**). Previous studies have reported that soil K commonly accumulates in surface or fertilized layers and decreases with increasing depth, which is consistent with the relatively limited vertical mobility of K in the soil profile (Mariele et al., 2021; Yin and Vyn, 2004). Therefore, the improved KUE and yield observed in this study may be associated with the ability of deep banding of CPS to maintain K availability within the active soybean root zone. Conventional PS is readily soluble and can release K rapidly after soil application, which may cause a temporary increase in soil available K beyond crop uptake demand. Part of the released K may then be fixed by soil colloids or transformed into less readily available soil K pools, thereby reducing short-term K availability and fertilizer use efficiency (Slaton et al., 2010). In contrast, the polymer coating of CPS enables slow, stable, and sustained K release, maintaining appropriate soil solution K concentrations throughout the critical soybean growth stages (**Supplementary Figure 2**). Combined with the deep banding method, this approach further reduces the contact area between fertilizer granules and bulk soil, effectively alleviating soil K fixation. In this study, DCPS increased soil AK content by 7.4% in 2023 and 7.0% in 2024 compared with BPS, providing a continuous and stable available K supply for soybean growth and development. Plant roots exhibit nutrient-induced growth, and root growth or distribution can be regulated by the spatial position of soil nutrient hot zones (Su et al., 2025; Wu et al., 2022). Previous studies have shown that 66% of the soybean roots are distributed in the top 20 cm of the soil, and root growth and root length density tend to increase in soil layers with good nutrient supply (Li et al., 2017b). Increased endogenous IAA and CTK accumulation and enhanced CAT and POD activities indicated active physiological responses of soybean roots to the improved nutrient supply under CPS and deep banding. These physiological improvements collectively alleviated oxidative stress and promoted robust crop growth, laying a solid physiological foundation for high soybean yield formation.

### 4.2 Soil microbial community responds to the different K fertilization schemes

Soil microorganisms serve as core drivers of terrestrial biogeochemical cycles, regulating soil nutrient transformation, maintaining soil fertility, and sustaining the stability of agroecosystems (Yang et al., 2020; Delgado-Baquerizo et al., 2016). Fertilization management represents a dominant anthropogenic factor that reshapes soil microbial diversity and community structure, further modulating microbe-mediated soil nutrient cycling processes (Ikoyi et al., 2020; Clausing et al., 2021). In accordance with previous findings, the present research demonstrated that K fertilization regimes significantly altered soil bacterial and fungal alpha diversity at the soybean pod-filling stage, with CPS application exerting a more pronounced promotional effect than conventional PS fertilization (**Figure 4**). These divergent microbial responses are mainly driven by fertilization-induced variations in soil physicochemical properties, which are widely recognized as the primary determinants of soil microbial community assembly (Palansooriya et al., 2025; Gao et al., 2022; Li et al., 2024).

Notably, fertilization treatments barely altered the composition of core bacterial and fungal phyla across the two experimental years, with no obvious divergence in high-level taxonomic structure among treatments. This finding is consistent with previous studies showing that fertilization-induced changes in soil microbial communities are often reflected by shifts in specific responsive taxa rather than by a complete restructuring of dominant high-level taxonomic composition (Li et al., 2017a; Shi et al., 2012). In the present study, significant treatment-specific differences were observed at the genus level. The DCPS treatment prominently enriched multiple beneficial bacterial and fungal genera closely associated with legume nutrient acquisition and soil nutrient cycling (**Figure 7**). The enriched core bacterial genera included *Aeromicrobium*, *Novosphingobium*, *Bradyrhizobium*, *Gaiella*, *Nitrospira*, and *Lysobacter*, while the key enriched fungal genera were *Psathyrella*, *Schizothecium*, *Rhizophagus*, *Funneliformis*, and *Panaeolus*.

The microecological optimization induced by DCPS fertilization was accompanied by significant increases in soil nitrogen and phosphorus-cycling enzyme activity, including urease, protease, ACP, and PDE. Mantel test and Spearman correlation analyses verified that the dominant microbial taxa’s abundances were positively and significantly correlated with soil inorganic nitrogen (IN), AK, and nutrient-cycling enzyme activities (*P* < 0.05). These tight ecological correlations originate from the functional traits of enriched beneficial microorganisms. Specifically, *Bradyrhizobium*, *Gaiella*, *Nitrospira*, and *Psathyrella* synergistically facilitate soil organic matter decomposition and carbon and nitrogen cycling. Among them, *Bradyrhizobium* can enhance soil K bioavailability via silicate mineral weathering, effectively supplementing the soil available K pool (Wei et al., 2024; Wang et al., 2015). Additionally, *Lysobacter* secretes extracellular hydrolases such as proteases and phosphatases, directly strengthening soil enzymatic catalytic capacity and accelerating nutrient mineralization (Zhao et al., 2021). The improved soil enzyme activity further elevates soil nutrient availability, forming a positive feedback loop that stimulates microbial metabolic activity and enriches beneficial microbial consortia. This interaction validates the general consensus that higher soil microbial diversity corresponds to stronger nutrient cycling capacity in agricultural soils (Zhang et al., 2020).

Soil microbial co-occurrence network analysis further showed that DCPS enhanced microbial network connectivity, as reflected by higher edge numbers, average degree, and average clustering coefficients. These results suggest that DCPS promoted a more closely connected microbial network. The connected network under DCPS indicated a more organized community structure with functionally differentiated ecological modules, which reduced functional redundancy and enhanced the stability and resilience of the soil microecosystem (Gao et al., 2022). Collectively, these results demonstrate that the integration of CPS and deep banding not only enriches beneficial microbial taxa but also constructs a more closely connected soil microbial network, thereby potentially enhancing soil nutrient cycling and supporting soil fertility maintenance.

### 4.3 Relationships between soil fertility, microbial community, enzyme activities, plant growth characteristics, and soybean productivity

As a core macronutrient for soybeans, K participates in the regulation of multiple plant physiological metabolisms and stress tolerance, and is a key determinant of final crop productivity (Pesini et al., 2025). The SEM in this study systematically clarified the complex interactive relationships among fertilization regimes, soil microbial properties, soil fertility, enzyme activities, plant physiological characteristics, and soybean yield. The SEM suggested potential pathways linking CPS application and deep banding with soybean productivity. The direct path coefficients from CPS application and deep banding to soybean productivity were relatively small, indicating that their effects were mainly mediated through indirect pathways involving microbial diversity, soil fertility, enzyme activity, and plant growth characteristics. Therefore, the agronomic benefits of DCPS should be interpreted as the combined outcome of multiple soil and plant processes rather than as a strong direct effect of fertilizer type or placement alone.

These findings support the consensus of previous agroecological studies that fertilization-induced changes in soil physicochemical properties reshape soil microbial communities, and in turn, microbe-mediated soil nutrient transformation further modulates crop growth and yield performance (Pradhan et al., 2025; Wei et al., 2024). The superior soybean productivity under DCPS was not merely attributed to increased soil IN and AK reserves but depended on the synergistic coupling of improved microbial community diversity, enhanced soil enzyme activity, and optimized plant physiological status. Deep banding of CPS effectively improved soil microecological conditions and enriched beneficial microbial taxa such as *Bradyrhizobium*. These microorganisms secrete organic acids to disrupt soil mineral structures, promote the dissolution of insoluble fixed K, and convert it into plant-available K, thereby continuously improving soil K supply capacity (Wang et al., 2015). Sufficient and stable K nutrient supply effectively promoted soybean N and K uptake and utilization, optimized plant growth status, and ultimately increased yield.

The improved crop performance under DCPS was closely associated with the enrichment of diverse beneficial microbial genera. *Aeromicrobium* and *Lysobacter* enhance soil enzyme activity and accelerate organic matter mineralization, thereby improving soil nutrient availability (Hoogstra et al., 2024; Zhao et al., 2021). *Rhizophagus* establishes arbuscular mycorrhizal symbiosis with soybean roots to facilitate the uptake of soil N, P, and K nutrients (Püschel et al., 2017). Moreover, *Funneliformis* and *Dominikia* enhance plant antioxidant capacity and alleviate oxidative damage to plant cells (Bilgili, 2025), which explains the significantly higher CAT and POD activities in soybean roots and leaves under DCPS treatment. In summary, the optimized K fertilization regime achieves coordinated improvements in soil microecology, soil fertility, and plant physiological performance, thereby realizing sustainable enhancement of soybean productivity.

## 5 Conclusion

The integration of controlled-release potassium sulfate application and deep banding fertilization effectively improved soybean productivity and nutrient use efficiency by comprehensively regulating soil microecological characteristics, soil nutrient status, and plant physiological traits. Across two consecutive field growing seasons, the controlled-release potassium sulfate combined with deep banding method treatment consistently outperformed potassium sulfate combined with broadcast incorporation method, potassium sulfate combined with deep banding method, and controlled-release potassium sulfate combined with broadcast incorporation method in terms of soybean yield, potassium use efficiency, and nitrogen use efficiency. Mechanistic analysis revealed that the superior agronomic performance of controlled-release potassium sulfate combined with deep banding method was driven by multiple synergistic beneficial factors: the enrichment of core beneficial microbial taxa represented by *Bradyrhizobium*, the optimization of soil microbial community structure and co-occurrence network stability, the enhancement of soil nitrogen and phosphorus cycling enzyme activities, the improvement of soil available nutrient pools, and the optimization of soybean antioxidant metabolism and endogenous hormone homeostasis. The coordinated improvements in soil microecological function, soil fertility, and plant physiological capacity collectively promoted significant soybean yield enhancement. This study systematically clarifies the regulatory mechanism of combined controlled-release potassium sulfate and deep banding method on soybean yield formation from the integrated perspectives of soil microecology and plant physiology. The findings provide a solid theoretical basis and feasible technical reference for optimizing potassium fertilization management, improving soybean nutrient utilization efficiency, and facilitating green and sustainable soybean production in the North China Plain.

## Data Availability

The datasets presented in this study can be found in online repositories. The names of the repository/repositories and accession number(s) can be found in the article/**Supplementary Material**.

## Glossary

ACP: acid phosphatase
AK: available potassium
ANOVA: analysis of variance
ASV: amplicon sequence variant
BCPS: controlled-release potassium sulfate combined with broadcast incorporation
BPS: potassium sulfate combined with broadcast incorporation
CAT: catalase
CPC: controlled-release potassium chloride
CPS: controlled-release potassium sulfate
CTK: cytokinin
DAS: days after sowing
DCPS: controlled-release potassium sulfate combined with deep banding
DPS: potassium sulfate combined with deep banding
ELISA: enzyme-linked immunosorbent assay
GFI: goodness-of-fit index
IAA: indole-3-acetic acid
IN: inorganic nitrogen
ITS: internal transcribed spacer
KUE: potassium use efficiency
NKF: potassium-free fertilization
NNF: nitrogen-free fertilization
NUE: nitrogen use efficiency
PC: potassium chloride
PCoA: principal coordinate analysis
PCR: polymerase chain reaction
PDE: phosphodiesterase
POD: peroxidase
PS: potassium sulfate
QIIME 2: Quantitative Insights Into Microbial Ecology 2
RMSEA: root mean square error of approximation
SEM: structural equation modeling
SRA: Sequence Read Archive
