# Supplementary material for: Controlled-release potassium sulfate combined with the deep banding method on soybean productivity and soil microbial characteristics
Source: Front Plant Sci. 2026 Jul 29;17:1904680. doi: 10.3389/fpls.2026.1904680 (PMC13461637; doi:10.3389/fpls.2026.1904680)
Supplement: Supplementary file 1 [file DataSheet1.pdf]

Supporting Information for

Controlled-release potassium sulfate combined with deep banding method on soybean productivity and soil microbial characteristics

Qianhui Chen†, Shuhan Yin†, Changzhi Li, Jie Qu, Yongxiang Gao, Qi Chen, Yanli Liu, Chengliang Li* and Zhaoming Qu*

National Engineering Research Center for Efficient Utilization of Soil and Fertilizer Resources, College of Resources and Environment, Shandong Agricultural University, Tai'an, China

***Correspondence:**

Chengliang Li

chengliang_li11@163.com

Zhaoming Qu

quzhaoming0504@163.com

Qianhui Chen† and Shuhan Yin† contributed equally to this work and should be considered co-first authors.

Keywords: Potassium fertilizer, Fertilization method, Soybean yield, Potassium use efficiency, Endogenous hormone

Captions of listed tables

Table. S1 Three-way ANOVA analysis

Table. S2 Topological parameters of bacterial and fungal co-occurrence networks at the genus level.

Table S1. Three-way ANOVA analysis

Index	Y (Year)	F (Fertilization)	B (Banding)	Y×F	Y×B	F×B	Y×F×B
Yield	*P*=0.000***	*P*=0.000***	*P*=0.639ns	*P*=0.023*	*P*=0.445ns	*P*=0.000***	*P*=0.108ns
KUE	*P*=0.014*	*P*=0.000***	*P*=0.235ns	*P*=0.947ns	*P*=0.911ns	*P*=0.019*	*P*=0.696ns
NUE	*P*=0.868ns	*P*=0.001***	*P*=0.184ns	*P*=0.680ns	*P*=0.267ns	*P*=0.896ns	*P*=0.264ns
IN	*P*=0.000***	*P*=0.007**	*P*=0.915ns	*P*=0.676ns	*P*=0.660ns	*P*=0.017*	*P*=0.362ns
AK	*P*=0.413ns	*P*=0.000***	*P*=0.930ns	*P*=0.268ns	*P*=0.328ns	*P*=0.005**	*P*=0.398ns
Protease	*P*=0.550ns	*P*=0.000***	*P*=0.000***	*P*=0.001**	*P*=0.008**	*P*=0.000***	*P*=0.021*
Urease	*P*=0.000***	*P*=0.000***	*P*=0.274ns	*P*=0.156ns	*P*=0.636ns	*P*=0.000***	*P*=0.016*
Acid phosphatase	*P*=0.000***	*P*=0.000***	*P*=0.584ns	*P*=0.009**	*P*=0.361ns	*P*=0.001**	*P*=0.219ns
Phosphodiesterase	*P*=0.006**	*P*=0.000***	*P*=0.164ns	*P*=0.397ns	*P*=0.703ns	*P*=0.000***	*P*=0.189ns
Root IAA	*P*=0.000***	*P*=0.000***	*P*=0.406ns	*P*=0.504ns	*P*=0.683ns	*P*=0.038*	*P*=0.745ns
Leaf IAA	*P*=0.000***	*P*=0.000***	*P*=0.362ns	*P*=0.307ns	*P*=0.474ns	*P*=0.003**	*P*=0.581ns
Root CTK	*P*=0.000***	*P*=0.000***	*P*=0.187ns	*P*=0.696ns	*P*=0.345ns	*P*=0.004**	*P*=0.396ns
Leaf CTK	*P*=0.000***	*P*=0.000***	*P*=0.385ns	*P*=0.027*	*P*=0.711ns	*P*=0.000***	*P*=0.430ns
Leaf CAT	*P*=0.000***	*P*=0.000***	*P*=0.301ns	*P*=0.003**	*P*=0.414ns	*P*=0.003**	*P*=0.137ns
Root CAT	*P*=0.003**	*P*=0.000***	*P*=0.861ns	*P*=0.006**	*P*=0.805ns	*P*=0.001**	*P*=0.516ns
Leaf POD	*P*=0.000***	*P*=0.000***	*P*=0.035*	*P*=0.935ns	*P*=0.756ns	*P*=0.001***	*P*=0.510ns
Root POD	*P*=0.000***	*P*=0.000***	*P*=0.082ns	*P*=0.909ns	*P*=0.501ns	*P*=0.001***	*P*=0.981ns

Notes: Values are P values from three-way ANOVA. Y, F, and B represent year, fertilizer type, and placement method, respectively. *p* values are retained to three decimal places. *, **, and *** indicate $p<0.05$, $p<0.01$, and $p<0.001$, respectively; ns indicates no significant difference.

Table S2. Topological parameters of bacterial and fungal co-occurrence networks at the genus level.

Microbial	year	Treatments	Number of nodes	Number of edges	Average degree	Graph Density	Modularity	Average clustering coefficient
Bacterial	2023	NKF	100	65	1.30	0.01	0.91	0.67
		DPS	100	480	9.60	0.10	0.58	0.79
		DCPS	100	762	15.24	0.15	0.43	0.80
	2024	NKF	100	71	1.42	0.01	0.93	0.75
		DPS	100	233	4.66	0.05	0.85	0.74
		DCPS	100	508	10.16	0.10	0.84	0.78
Fungal	2023	NKF	81	92	2.27	0.03	0.78	0.79
		DPS	100	308	6.16	0.06	0.39	0.83
		DCPS	99	433	8.75	0.09	0.64	0.85
	2024	NKF	81	166	4.10	0.05	0.84	0.74
		DPS	57	153	5.37	0.10	0.66	0.78
		DCPS	90	240	5.33	0.06	0.80	0.88

Captions of listed figures

Figure S1. Air temperature and Precipitation in 2023 (A) and 2024 (B)

Figure S2. Cumulative release curve of potassium from controlled-release potassium sulfate in water (25 °C), as determined by the electrical conductivity method.

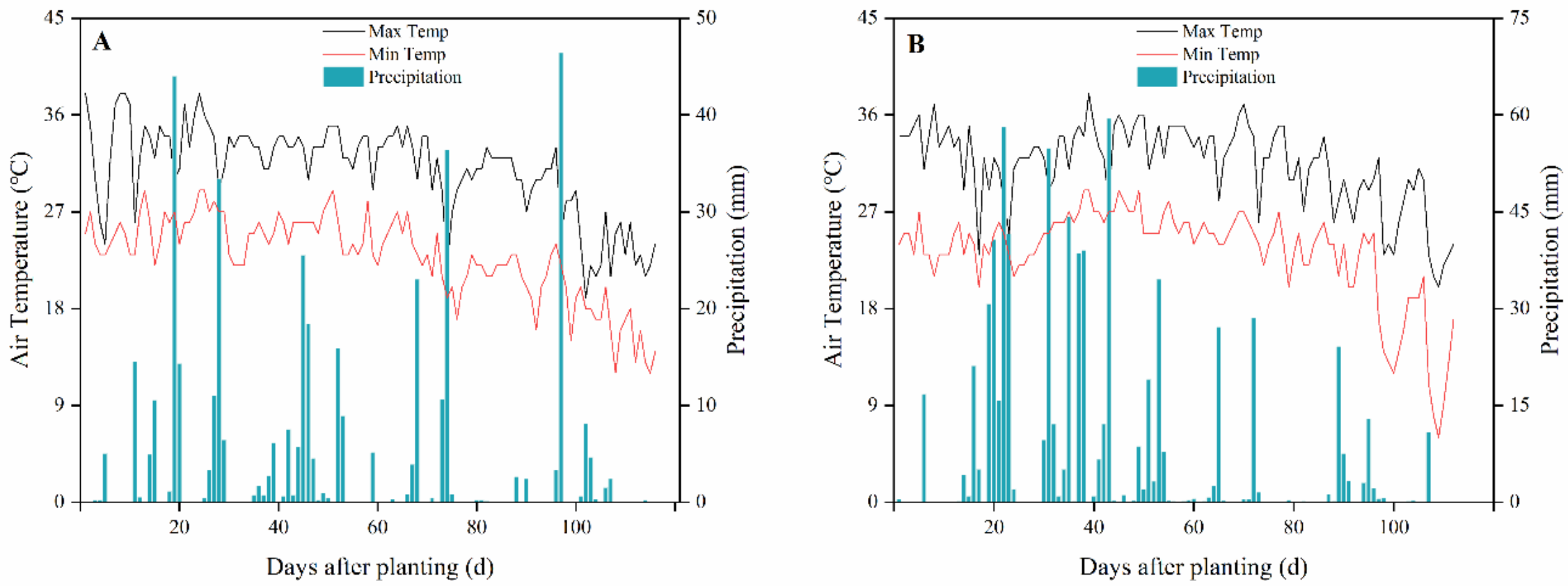

Figure S1. Air temperature and precipitation in 2023 (A) and 2024 (B)

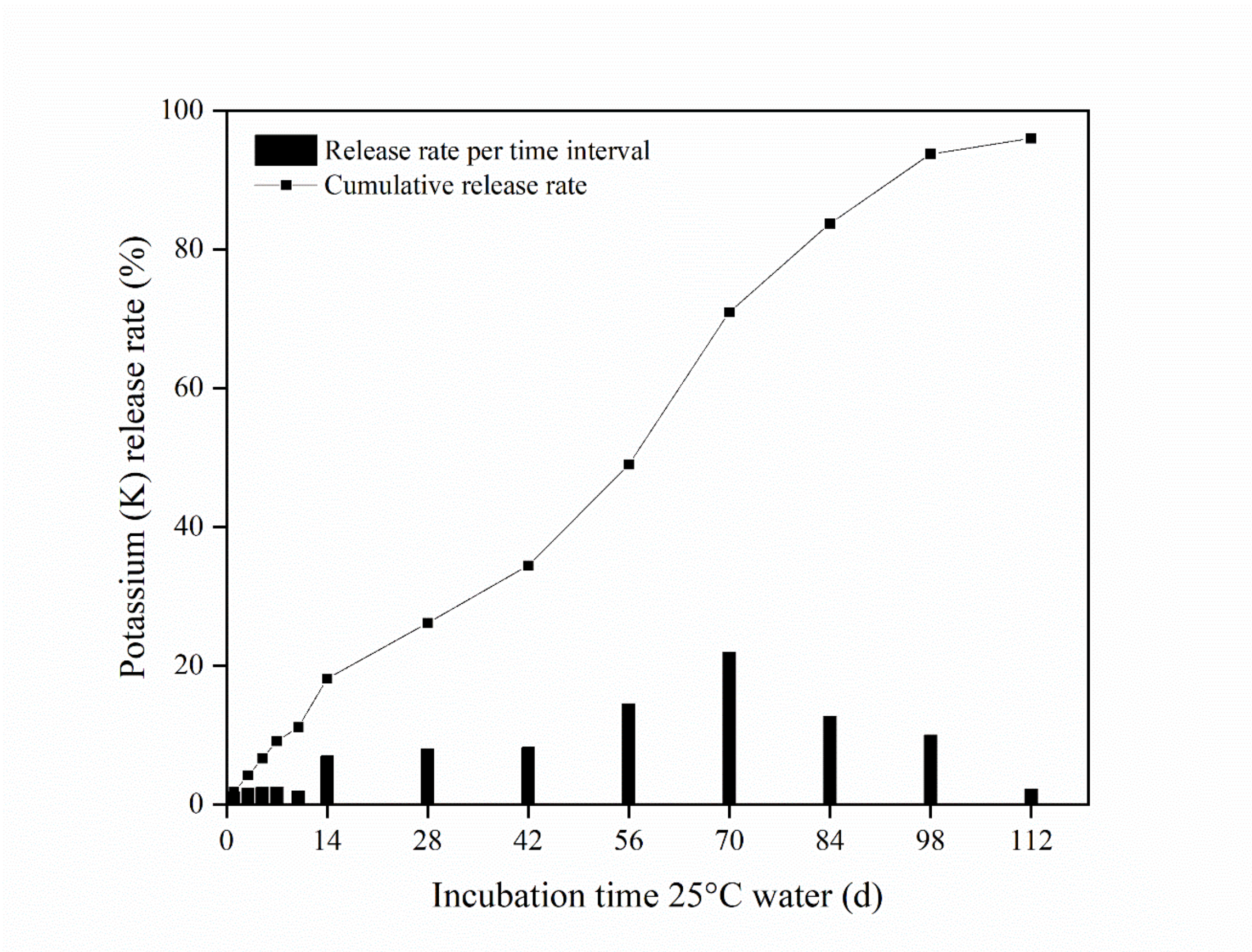

Figure S2. Cumulative release curve of potassium from controlled-release potassium sulfate in water (25 °C), as determined by the electrical conductivity method.
